# Epidemiology of Ocular Manifestations in Autoimmune Disease

**DOI:** 10.3389/fimmu.2021.744396

**Published:** 2021-11-02

**Authors:** Katie Glover, Deepakkumar Mishra, Thakur Raghu Raj Singh

**Affiliations:** School of Pharmacy, Medical Biology Centre, Queen’s University Belfast, Belfast, United Kingdom

**Keywords:** ocular manifestation, autoimmune disorders, retinopathy, epidemiology, systemic autoimmune disorders

## Abstract

The global prevalence of autoimmune diseases is increasing. As a result, ocular complications, ranging from minor symptoms to sight-threatening scenarios, associated with autoimmune diseases have also risen. These ocular manifestations can result from the disease itself or treatments used to combat the primary autoimmune disease. This review provides detailed insights into the epidemiological factors affecting the increasing prevalence of ocular complications associated with several autoimmune disorders.

## Introduction

Autoimmune diseases result from the body’s immune system attacking self-antigens (1) and are classified as either organ-specific or systemic depending on the target area within the body. Although there are over 80 known autoimmune diseases, the exact aetiology of many of these diseases is still unknown. Genetics and environmental factors are crucial in dictating disease susceptibility, prevalence and severity through the cellular immune system.

The wide variety of ocular manifestations associated with various autoimmune diseases are often overlooked, and their significance is underestimated. These manifestations range from minor disturbances to sight-threatening conditions that need immediate medical intervention. The eye is a delicate organ with a microenvironment sensitive to systemic changes within the body, which can also act as the first indicator of underlying autoimmune disease (2, 3). Ocular manifestations can also arise during active disease or years following diagnosis. Delaying treatment of these manifestations directly impacts a patient’s quality of life, and in some cases, there is undoubtable risk for visual impairment.

Every section of the eye is a potential target for autoimmune-related complications. Ideally, an ocular examination should become a routine part of disease management to diagnose, investigate and treat any arising ocular symptoms on time. Furthermore, the importance of regular screening, even for those who are asymptomatic, should be emphasised due to the potential for acute, sight-threatening ocular complications, which are observed with several of the autoimmune diseases covered within this review.

Autoimmune diseases are increasing globally, from an estimated prevalence of 3.2% between 1965 and 1995 to 19.1 ± 43.1 reported in 2018 (4, 5). By 2026 the global diagnosis market size for autoimmune diseases, currently worth $4.1B, is estimated to reach $6.3B (6). Some reasons for this increase can be owed to genetic predisposition in an ageing population and improved diagnostic techniques. However, increasing prevalence has been more greatly influenced by environmental factors, thus suggesting a reason these issues can be reduced.

In addition, polyautoimmunity or multiple autoimmune syndromes in a single patient is not uncommon, such as the association between rheumatoid arthritis, thyroiditis and type 1 diabetes mellitus (7). Such conditions increase the risk of systemic manifestations, including those affecting the eye.

With a global population seeing an increasing prevalence of autoimmune diseases, with additional risk of polyautoimmunity and an ageing population, we can only hypothesise the potential for accompanying ocular manifestations of these diseases also to increase. Various reviews have been published to highlight the prevalence of ocular manifestation of different autoimmune disorders, however, the review to highlight the epidemiological prevalence along with recent literature is highly desired (8–11). This review aims to raise awareness of the various ocular complications associated with autoimmune diseases and those at greatest risk so that particular care can be taken with screening and diagnosis.

## 2 Autoimmune Disorders and Their Ocular Effects

**Figure 1:**
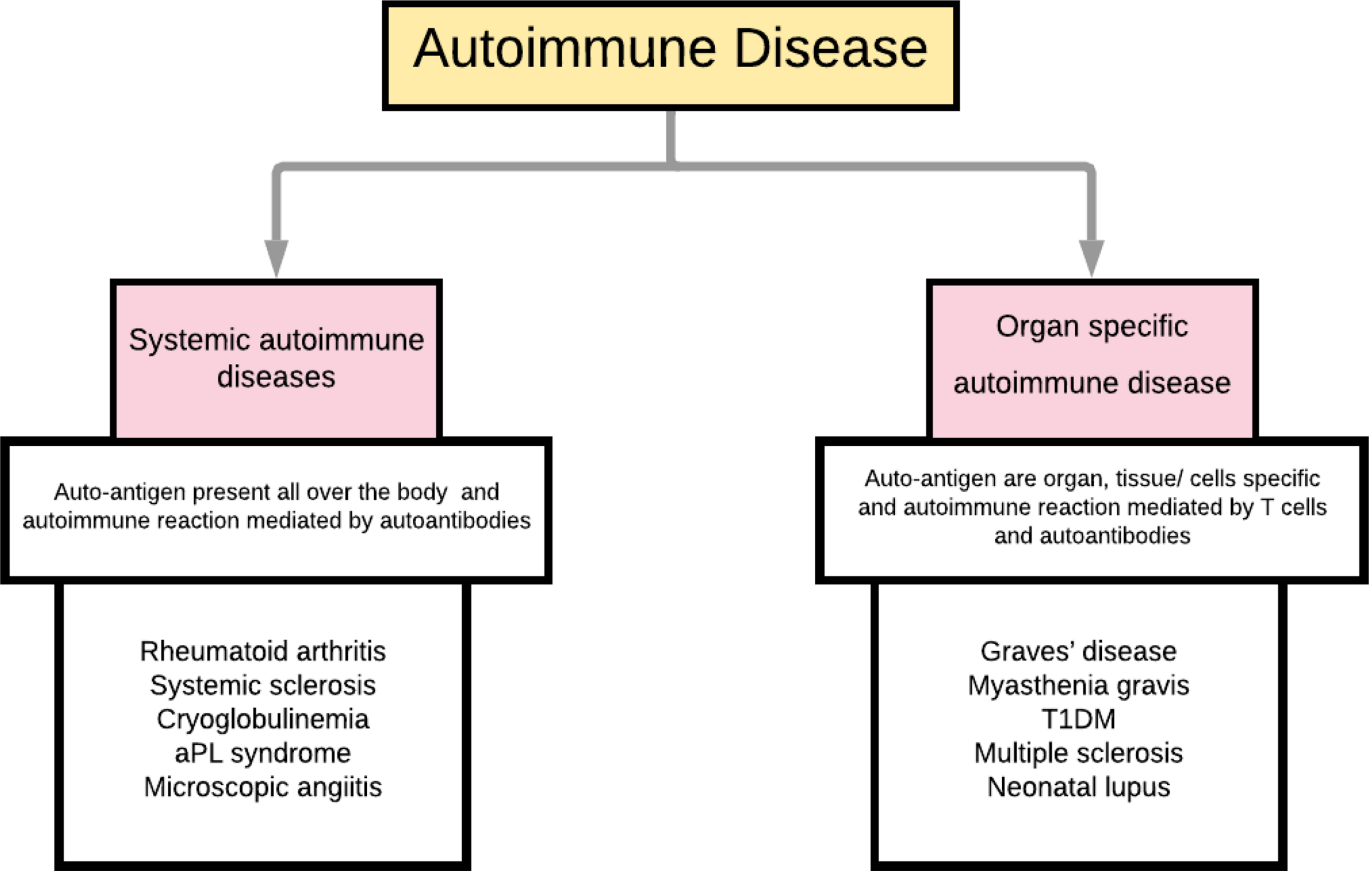
Classification of autoimmune diseases.

**Figure 2:**
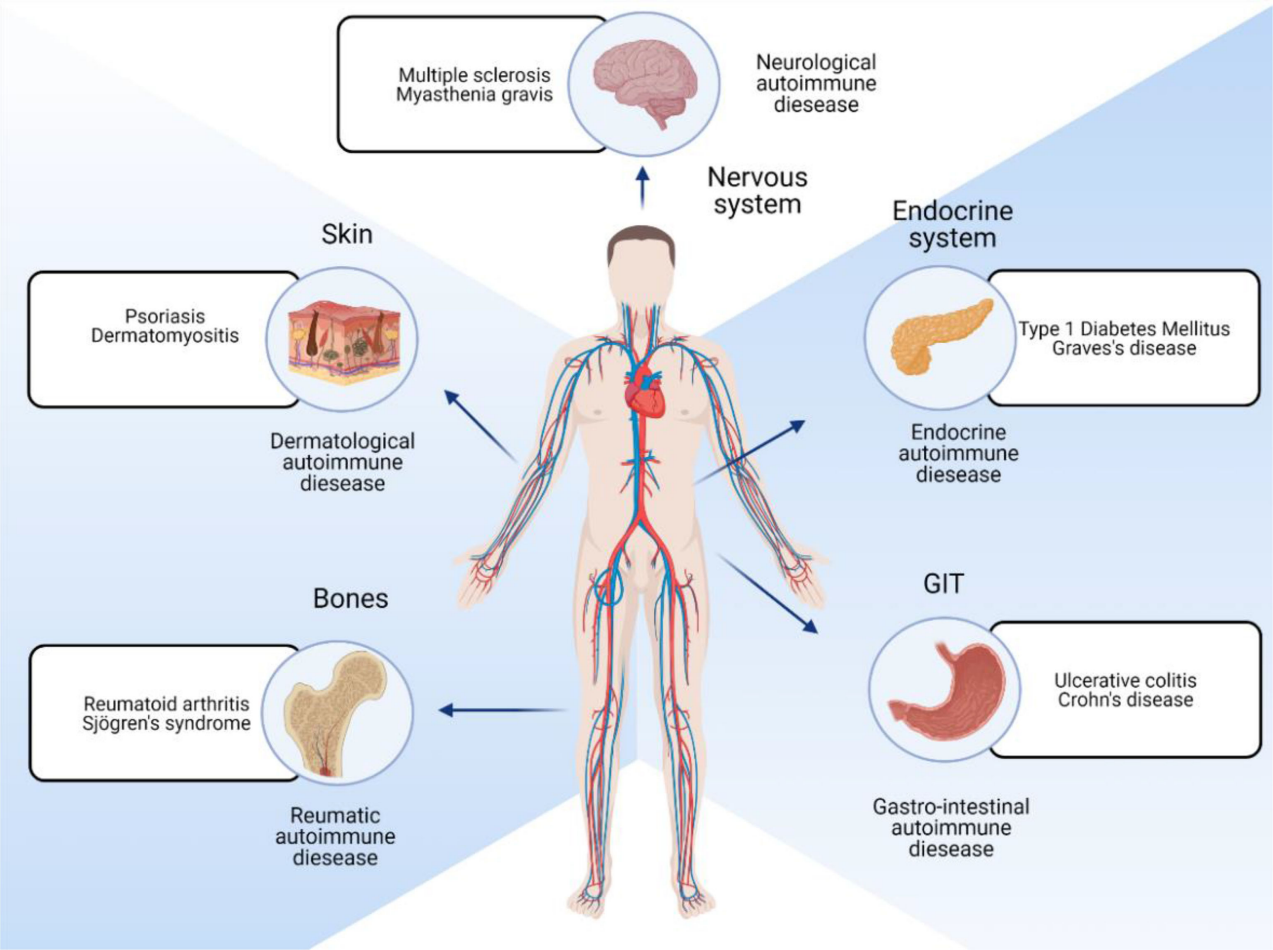
Different locations of the body that are affected by autoimmune diseases.

Autoimmune diseases can be broadly classified as systemic and organ-specific (**Figure 1**). Within systemic autoimmune disorders, autoimmunity unanimously targets the ubiquitously (universally) expressed self-antigen and leads to antibody-mediated end-organ injury. Autoimmune diseases also affect different parts of the body, including the skin and gastrointestinal system (**Figure 2**). Autoantibodies play a significant role in systemic autoimmune disorders compared to T cells. In organ-specific autoimmune disorders, the autoantibodies and the T cells target the organ/tissue/cell-specific antigens leading to a specific and pointed autoimmune reaction.

Ocular complications resulting from autoimmunity ranges from less serious effects, e.g. topical conjunctivitis and scleritis, to more chronic and serious effects, e.g. neuropathies and choroidal angiogenesis. As summarized in **Table 1**, a wide range of autoimmune diseases are often associated with common ocular manifestations; however, some ocular manifestations are diseases specific (**Table 2**). Various autoimmune diseases discussed in this review are related to adverse ocular manifestations; further detail on the prevalence and the symptoms of ocular effects of multiple classes of autoimmune diseases are discussed.

**Table 1:** Common ocular manifestations of various autoimmune diseases.

Common Ocular manifestations Disease	Keratoconjunctivitis sicca	Scleritis	Episcleritis	Retinal vasculitis	Conjunctivitis	Blepharitis	Keratitis	Uveitis	Macular oedema	Choroiditis	Photophobia	Retinal detachment	Vitreal and retinal haemorrhage	Retinal vein occlusion	Glaucoma	Corneal ulcers	Diplopia	Optic atrophy	Cataracts
Rheumatoid Arthritis	✓	✓	✓	✓	✓	✓	✓	✓	✓	✓		✓							
Sjogren’s Syndrome	✓	✓	✓	✓				✓			✓					✓			
Behcet’s Disease				✓				✓	✓			✓	✓	✓	✓			✓	
Crohn’s Disease	✓	✓	✓	✓	✓	✓		✓								✓			✓
Ulcerative Colitis		✓	✓					✓				✓		✓					
Celiac Disease	✓				✓		✓	✓						✓		✓			✓
Multiple Sclerosis								✓				✓	✓		✓				✓
Guillain-Barre Syndrome	✓								✓		✓		✓				✓		
Myasthenia Gravis	✓					✓											✓		
Type 1 Diabetes Mellitus									✓						✓				✓
Graves’ disease	✓														✓		✓		
Hashimoto’s Thyroiditis	✓						✓				✓						✓		
Psoriasis	✓				✓	✓													✓
Systemic Sclerosis	✓											✓					✓		✓
Dermatomyositis			✓										✓	✓			✓	✓	✓

**Table 2:** Rare ocular manifestations of various autoimmune diseases.

Autoimmune Disease	Uncommon ocular manifestations associated with the autoimmune disorders
Rheumatoid Arthritis	Peripheral ulcerative, pterygium, peripheral stromal thinning, acute central corneal melting, choroiditis,
Sjogren’s Syndrome	Retinal vasculitis, corneal melt, corneal perforation, conjunctival epithelial keratinization, optic neuropathy, sterile corneal ulcer.
Behcet’s Disease	Hypopyon, papillitis, chorioretinitis, retinal periphlebitis, periarteritis, posterior synechiae, iris bombe, keratic precipitates, epiretinal membrane.
Crohn’s Disease	Subepithelial infiltrates perivascular sheathing, lid swelling, lid margin, orbital myositis, optic neuritis, dyacroadenitis, palpebral ptosis, choroidal neovascularization, central serous chorioretinopathy.
Uclerative Colitis	Orbital swelling, vasculitis, iritis, central serous chorioretinopathy, keratopathy, uveal effusion, choroidal neovascularisation, cranial nerve palsy, optic neuritis.
Celiac Disease	Orbital myositis, keratomalacia, microbial keratitis, retinopathy, pseudotumor cerebri, nyctalopia, thyroid-associated orbitopathy.
Multiple Sclerosis	Cranial nerve palsies, optic neuritis, optic neuritis, internuclear ophthalmoplegia, nystagmus, pars planitis, retinal periphlebitis, oscillopsia, reduced colour perception, lesions affecting the chiasm.
Guillain-Barre Syndrome	Accommodation insufficiency, papillophlebitis, cotton wool spots, pupillary dysfunction, ophthalmoparesis, esotropia, lagophthalmos, ectropion, corneal sensitivity, papilloedema, Colgan’s lid twitch, mydriasis, vertical gaze palsy,
Myasthenia Gravis	Internuclear ophthalmoplegia, ptosis, thyroid eye disease, ophthalmoparesis, lagophthalmos, orbicularis weakness,
Type 1 Diabetes Mellitus	Diabetic retinopathy, accommodation insufficiency, reduced vascular density, corneal erosion, corneal hypoesthesia
Graves’ Disease	Thyroid-associated ophthalmology, corneal hysteresis, proptosis, meibomian gland dysfunction, ocular lesions, conjunctival erythema, eyelid oedema
Hashimoto’s Thyroiditis	Thyroid-associated ophthalmology, ptosis, reduced color vision, upper eyelid retraction, chemosis, conjunctival prolapse, roptosis, exophthalmos, lid lag,
Psoriasis	Eyelid psoriasis, conjunctival lesions, xerosis, ectropion, orbital myositis, corneal pigment dispersion, corneal opacities, ptosis, peripheral corneal melt syndrome, meibomian gland dysfunction,
Systemic Sclerosis	Telangiectasia, keratoconus, iris transillumination, meibomain gland dysfunction, keratopathy, corneal astigmastism
Dermatomyositis	Ptosis, strabismus, conjunctival oedema, nystagmus, iritis, cotton wool spots, internuclear ophthalmoplegia, papilloedema, orbital myositis, canthal scars

### Rheumatic Autoimmune Diseases

Autoimmune rheumatic diseases (ARDs) are a diverse group of conditions that primarily affect the joints, bones, muscle, and connective tissue, with rheumatoid arthritis being the most common.

#### Rheumatoid Arthritis

*Rheumatoid arthritis* is a chronic disease causing systemic polyarthritis, generally in a bilateral form, characterised by inflammation of the synovium tissue. In recent years, global prevalence estimates have ranged from 1 – 2% (12, 13).

Keratoconjunctivitis sicca, or dry eye disease, is the most common ocular presentation of rheumatoid arthritis, occurring in 10 – 35% of patients. Other common manifestations include episcleritis, scleritis, peripheral ulcerative keratitis (PUK) and retinal vasculitis. In a recent study, a third of rheumatoid arthritis patients displayed ocular manifestations, with keratoconjunctivitis sicca accounting for 85% of ocular conditions (14). Dry eye disease results from damage to the lacrimal gland by attacking B and T lymphocytes. Although no effective treatment currently exists, lubricating eye drops can be used to relieve and manage the symptoms of itchiness, redness and foreign body sensation that sufferers experience. In more severe cases, occlusion of lacrimal drainage puncta or tarsorrhaphy may have to be utilised. No correlation has been found between disease severity with dry eye disease severity in rheumatoid arthritis patients. Rather more severe dry eye was associated with a longer disease duration (15). Corneas of rheumatoid arthritis patients have been found to be significantly thinner than healthy control cohorts, which decreased with increasing corneal curvature (16).

Episcleritis is present in up to 10% of rheumatoid arthritis patients, resulting from inflammation of the suprachoroidal layer. Topical eye drops can be administered to constrict blood vessels; however, these will not target vessels deep in the sclera, presenting problems for effective treatment. Thinner choroid layers and increased resistance to blood flow in rheumatoid arthritis patients were found in comparison to healthy control groups (17), however, no correlation with disease severity was detected, indicating the risk of potentially severe ocular manifestations in well-managed rheumatoid arthritis patients.

Scleritis accounts for 10% of ocular complications in rheumatoid arthritis patients (12), but can be considerably more painful. PUK is rare in rheumatoid arthritis, mainly reported in case studies. However, one case highlights rapid development from generalised symptoms to severe bilateral PUK involving vision loss (18). Scleritis and PUK have the potential to develop into retinal vasculitis (19), which can result in vision loss if not intervened within a timely manner. In some cases, retinal vasculitis is asymptomatic, reinforcing regular screening of all patients to be paramount in identifying and treating ocular manifestations.

#### Sjogren’s Syndrome

*Sjogren’s syndrome* is a chronic inflammatory disease, laying particular attack to the lacrimal and salivary glands. Patients with Sjogren’s syndrome will present sicca symptoms due to inflammatory damage of lacrimal and salivary exocrine glands. Recent global prevalence is estimated to be up to 1% of the population (20).

1 in 3 patients with Sjogren’s syndrome shows ocular manifestations, with 13% of this being sight-threatening (21). Like rheumatoid arthritis, dry eye disease is the most common ocular manifestation. Acting as a precursor, 1 in 10 American patients over the age of 50 diagnosed with dry eye disease ultimately have underlying Sjogren’s syndrome (22). Unfortunately, for those that are diagnosed, the time between the first presentation of ocular symptoms and Sjogren’s syndrome diagnosis is estimated to be 10 years (21), indicating the lack of awareness of the significance of ocular manifestations within Sjogren’s syndrome.

Other conditions such as episcleritis, scleritis, retinal vasculitis and corneal melt or perforation are commonly associated with primary Sjogren’s syndrome, with some research indicating males may be at more risk of developing these ocular manifestations (23), although this finding can be challenged (24).

#### Bechet’s Disease

*Bechet’s Disease* is characterised by chronic vasculitis of several organs, including the eyes and nervous system. Bechet’s disease has a high incidence of ocular manifestations, with 70% of patients haven being found to show some form of the ocular disorder (25). With Bechet’s disease, ocular symptoms are generally not the first to manifest, however, their significance cannot be underestimated.

Recurrent bilateral uveitis is prevalent in approximately two-thirds of Bechet’s disease patients (26), with 25% of these patients prone to developing blindness (27). In addition, serious complications can occur even in an early stage of Bechet’s disease-associated uveitis, such as retinal lesions (75 -80%) (28), cataracts (39.5%) and secondary glaucoma (17.1%) (29), leading to visual impairment.

One male patient who presented decreased visual acuity and intraretinal haemorrhages alongside macular oedema and disc oedema was given a trial of systemic corticosteroids, immunosuppressants and a dexamethasone intravitreal implant. Although macular oedema resolved, decreased visual acuity remained, conveying the irreversible effects of Bechet’s disease-associated uveitis (25). In one study, macular oedema occurred in over half of patients. However retinal periphlebitis was most common (29).

### Gastrointestinal Autoimmune Diseases

Gastrointestinal autoimmune diseases involve immune attacks focused on organs of the gastrointestinal tract. The current lack of awareness surrounding these diseases is evident through literature case studies. For example, there has been limited research into how the cornea is affected in IBD patients. Recent research, however, found Crohn’s disease patients who showed no symptoms of ocular involvement were observed to have reduced corneal thickness and reduced tear quantity (30).

#### Crohn’s Disease

*Crohn’s disease* is characterised by flares of inflammation in the gastrointestinal tract leading to scarring and ulceration. Crohn’s disease can affect any age group but appears most common in the late teens. Recent studies indicate an increase in prevalence (31).

**Figure 3:**
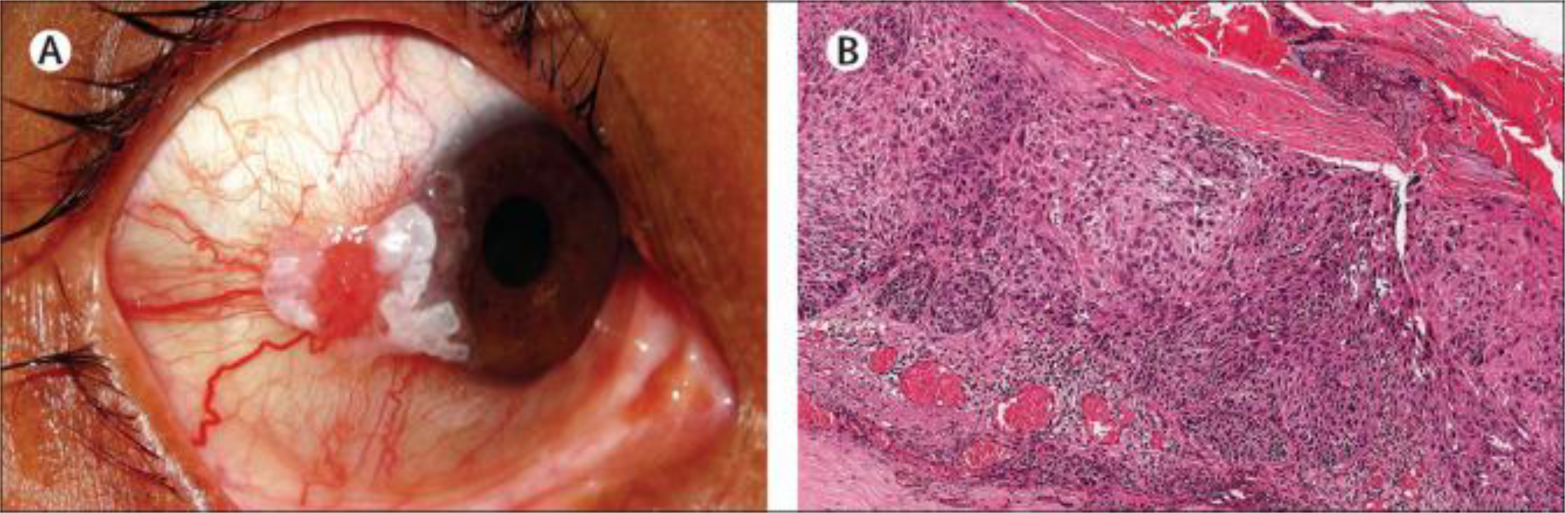
**(A)** Observation of limbal ocular surface squamous neoplasia along with keratin plaque in patient suffering from Crohn**’**s disease **(B)** Histopathology of invasive squamous cell carcinoma post excision of the tumour using hematoxylin and eosin stain. Image used with permission from Triphathy et al. (35).

Up to 12% of Crohn’s disease patients show ocular manifestations, with episcleritis being the most common, closely followed by scleritis and uveitis (32). Posterior uveitis in Crohn’s disease has a reported incidence of 5.6 people per 100,000 population (33). Oral prednisolone is reported to alleviate ocular lesions, mild vitritis and intraretinal haemorrhages in a patient occurring before Crohn’s disease diagnosis, with no reports of permanent ocular damage (34). Ocular neoplasia has also been linked to Crohn’s disease (**Figure 3**) (35).

Ocular manifestations have appeared primarily in the early stages of the disease (36), but can also occur during active disease or remission. For example, orbital myositis, although still rare in Crohn’s disease, has been reported more frequently than in ulcerative colitis. One patient presented acute onset of orbital myositis during a period of disease remission, which was quickly resolved with prednisolone and adalimumab treatment (37).

Less common manifestations include “corneal ulcers, blepharitis, cataracts, conjunctivitis, macular haemorrhage, subepithelial infiltrates, perivascular sheathing and retinal vasculitis” (36). Dry eye disease is more common in Crohn’s disease when compared to ulcerative colitis (30, 38). Retinal vasculitis was detected 5 years before gastrointestinal symptoms allowed Crohn’s disease diagnosis (2).

#### Ulcerative Colitis

*Ulcerative colitis* is another form of IBD only affecting the colon and rectum. Despite the increasing prevalence, the exact aetiology of UC remains unknown.

Ulcerative colitis patients also see a reported incidence of ocular manifestations between 4 – 12%, although uveitis and iritis are more commonly associated with ulcerative colitis than Crohn’s disease (32). One study found 83% of patients to have ocular manifestations, with cataracts and conjunctivitis being the most frequent (38). Contradictory data exist, with some studies reported higher levels of ocular manifestations for ulcerative colitis (39, 40) and one study reporting Crohn’s disease patients to be at higher risk of developing ocular manifestations (41).

Other rare manifestations include central retinal vein occlusion, vasculitis, peripheral corneal ulcers, corneal infiltrates, central serous chorioretinopathy and retinal detachment. Keratopathy and night blindness resulting from Vitamin A deficiency can also occur (42). Less frequently encountered ocular side effects include vitritis, uveal effusion, choroidal neovascularisation, cranial nerve palsy, and optic neuritis. Although more common in Crohn’s disease, case reports of orbital myositis associated with ulcerative colitis exist (43, 44). In addition, case reports of ulcerative colitis patients with CRVO have been reported, in some cases leading to permanent vision loss (2, 45). Orbital swelling has been reported in an ulcerative colitis patient, providing the first known association of ulcerative colitis and IgG4-ROD (46).

#### Celiac Disease

*Celiac disease* is a chronic disease of the small intestine affecting 1 – 2% of the global population (47), and is triggered by long-term ingestion of gluten. Moreover, up to 15% of celiac patients have Sjogren’s syndrome as a secondary autoimmune disease (48), increasing the risk of ocular problems.

Vitamin D deficiencies occur in 20 - 60% of celiac patients (47), leading to potential cataract formation. In addition, celiac patients are at greater risk of developing cataracts when compared to the general population due to disease factors such as oxidative stress and dehydration due to chronic diarrhoea. Vitamin A deficiency can also occur in up to a third of patients, leading to nyctalopia, dry eye disease, corneal ulcers (49).

Ocular manifestations can indicate asymptomatic celiac disease (50), including filamentary keratitis, keratomalacia, microbial keratitis, corneal ulcers and diplopia. Diplopia is more common in female patients who should then be investigated for orbital myositis. Reduced tear film functions and abnormalities in the corneal epithelium structures also occur in celiac disease patients (51). Retinopathy has been associated with celiac disease, especially when linked to type 1 diabetes mellitus (52). Celiac disease has been associated with anterior scleritis (53), where ocular and gastrointestinal symptoms coincided following gluten consumption, which was alleviated once a gluten-free diet was resumed.

Most cases of celiac remain undetected due to asymptomatic cases and poor disease awareness, which can have potentially devastating effects on the eye. Dogan et al. observed thinner subfoveal choroid layers in celiac patients who took more than 60 months to diagnose, indicating the need for more efficient diagnosis and awareness to prevent ocular complications from developing (54).

### Neurological Autoimmune Diseases

Neurological autoimmune diseases, such as multiple sclerosis, result from an immune-driven inflammatory attack on the central nervous system, resulting in severe disability and systemic manifestations.

#### Multiple Sclerosis

*Multiple sclerosis* is a progressive disease affecting the central nervous system, leading to severe disability. The autoimmune reaction leads to demyelination of nerve fibres, further leading to reduced or prohibited transmission of signals along the nerves. Studies suggest ocular movement is affected in 70% of MS cases (55). Multiple sclerosis prevalence is increasing, with 2 million cases globally (56).

Optic neuritis is a common manifestation occurring to due optic nerve lesions and affecting 7 out of 10 multiple sclerosis patients (57), especially in females. Optic neuritis is generally characterised by acute vision loss and ocular pain; however, severe complications are also reported to exist. These complications, or atypical optical neuritis, involve retinal haemorrhages, optic atrophy and a swollen optic nerve and require intensive treatment to prevent vision loss or chronic optic neuritis.

Pupillary disturbances have also been reported in MS, with approximate prevalence in 60% of multiple sclerosis patient cohorts (58). In addition, internuclear ophthalmoplegia occurs in 30% of multiple sclerosis cases and causes reduced eye movement due to lesions on the medial longitudinal fasciculus (59). Nystagmus is also common in multiple sclerosis patients, with acquired pendular nystagmus (APN) being the most common (60). APN can cause severe visual impairment; however, this can be reversed with timely and appropriate treatment (61).

The occurrence of multiple sclerosis-associated uveitis varies in the literature, with the highest known predictions reported at 36% (62). If overlooked, multiple sclerosis-associated uveitis can result in cataracts, glaucoma, cystoid macular oedema or retinal detachment.

#### Gullain-Barre Syndrome

With an estimated incidence of 1 in 100,000 population (63), Gullain-Barre syndrome causes demyelation and axonal degeneration, typically following preceding infection of the gastrointestinal or respiratory tract. Despite various subtypes, Gullain-Barre syndrome is underpinned by deteriorating nerve conduction and limb and facial muscle weakness. Symptoms progress rapidly, often reaching peak worsening of disease in a matter of weeks.

Accommodation insufficiency and ptosis, even in the absence of ophthalmoplegia, can indicate underlying Gullain-Barre syndrome (64). Papillophlebitis has also been documented as an initial disease manifestation that can lead to serious complications, such as haemorrhage of the optic nerve, macular oedema and cotton wool spots (65).

Up to 50% of patients have a form of cranial nerve involvement (64), and ophthalmoparesis is a common ocular manifestation of Gullain-Barre syndrome. Palsies of cranial nerves, commonly the third, sixth and twelth, can result in symptoms such as esotropia, ocular pain, ocular muscle paresis, corneal sensitivity, lagophthalmos, ectropion, reduced eyelid movement and dry eye disease (66). Papilloedema can occur due to increased cerebral fluid and cerebral oedema in some Gullain-Barre patients (67).

Due to similarities in clinical symptoms, such as lid abnormalities and pupillary dysfunction, Miller Fisher Syndrome (MFS) can be mistaken for ocular myasthenia gravis (68), delaying appropriate treatment of ocular manifestations. MFS accounts for 5 to 10% of Gullain-Barre cases and is characterised by diplopia, usually absent in Gullain-Barre syndrome, due to external ophthalmoplegia (69). MFS is more prominent in Asia, accounting for up to a quarter of Gullain-Barre cases in Japan alone (70). Additional ocular manifestations of Gullain-Barre syndrome include blepharoptosis, blurred vision, photophobia, vertical gaze palsy, internal ophthalmoplegia, abnormal lid function, mydriasis, anisocoria, ptosis, lid retraction, upper lid jerks, lid nystagmus and lagophthalmos. Colour-blindness, decreased visual acuity, pupillary abnormalities, and supranuclear gaze palsy have also been reported.

#### Myasthenia Gravis

*Myasthenia gravis* is a chronic disease resulting in reduced signal transmission in skeletal muscles due to the destruction of receptor cells at nerve junction. As a result, myasthenia gravis patients are vulnerable to developing thyroid disorders, further increasing the risk of thyroid eye disease (71).

The prevalence of myasthenia gravis is increasing, with global prevalence currently estimated to be 20 people per 100,000 population (72). Notably, one study found an ocular weakness to be the initial symptom in 82% of patients studied (73).

Diplopia and asymmetric extraocular involvement (74, 75) are common first symptoms of myasthenia gravis, suggesting ocular muscle involvement known as ocular myasthenia gravis (OMG). Currently, OMG occurs in 10 – 40% of myasthenia gravis sufferers (76). These patients will experience diplopia, ptosis, pupil sparing ophthalmoplegia, internuclear ophthalmoplegia and thyroid eye disease. Ophthalmoparesis can occur in all extraocular muscles, leading to severely restricted globe movement. In a cohort study by Tang et al., just under 70% and 25% showed diplopia and ptosis respectively on first evaluation. Of all 40 patients, the lateral rectus muscle was affected, leading to restriction of eye movement (73).

Dry eye disease has been reported in 21% of OMG patients (77), resulting from lagophthalmos and orbicularis weakness, while blepharitis is also associated (74). Although OMG is more common in myasthenia gravis, rare case reports confirm nystagmus and pseudo-intranuclear ophthalmoplegia (78). These patients will show limited adduction.

### Endocrine Autoimmune Diseases

In endocrine autoimmune disorders, hormone-producing glands are attacked by the immune system, resulting in the under-or over-production of various hormones needed to maintain homeostasis.

#### Type 1 Diabetes Mellitus

In *type 1 diabetes mellitus*, the destruction of insulin-producing beta-pancreatic cells results in an inability to control blood glucose levels since insulin signals glucose uptake into cells. Type 1 diabetes mellitus is a systemic disease that can lead to severe complications. Thus early detection is key to preventing organ damage, including the eye. Unlike type 2 diabetes, type 1 is often diagnosed earlier in life.

In 2020, the global prevalence of type 1 diabetes was 9.5%. However, in the UK alone, the prevalence of diabetes increased 40% between 1980 – 2014, with type 1 diabetes accounting for approximately 10% of these cases (79).

Diabetic retinopathy (DR), diabetic macular oedema (DME), cataracts and glaucoma have been reported in type 1 diabetes patients, with diplopia being more common in the early-stage disease (80). Lens changes, which occur naturally with ageing, are more pronounced in those with diabetes, in addition to lower accommodation reflexes (81). Vascular damage may not be detectable during the prediabetes period; thus, eye disease may have time to develop and progress before it is even identified in the absence of regular ophthalmic screening.

Early screening for DR is key for eyesight preservation, with evidence of early intervention reducing the risk of DR disease development (82) and the opportunity to prevent a fall in visual acuity (83). With a third of diabetes patients over 40 developing DR (84), implementing early and regular ophthalmic screening could prevent vision loss in millions of people. Hammes et al. found that 27.4% of a T1DM cohort developed retinopathy, with 8% severe cases. 40 year follow up estimates determined 84.1% to have retinopathy, 50.2% being severe cases, thus conveying the potential implications of lack of awareness, lack of screening, and untimely treatment on patients (85). Similarly, Srinivasan et al. found 43% of the studied cohort experienced worsening DR upon follow up (86).

While decreased visual acuity is greatly associated with the degree of capillary loss in patients with DR, these changes can be asymptomatic. This is supported by the findings of Duet al.t al, whereby patients who underwent OCT-A and OCT imaging, however approximately 30% showed vascular density reductions in the deep capillary plexus, despite showing no symptoms of DR (87). This conveys the importance of early screening for all patients.

Type 1 diabetes mellitus patients are twice as likely to develop glaucoma and cataracts when compared to healthy individuals (80), with a higher incidence rate for African American populations (88). In addition, females are at greater risk of cataracts related to type 1 diabetes (89, 90). Occurrence of cataracts are also reported in 0.7 – 3.5% of paediatric type 1 diabetes cases (91), often within the first 6 months of diagnosis (92) and can lead to vision loss. Given this, regular ophthalmic evaluation should be conducted even in those showing no obvious cataract symptoms. However, there is a lack of clear information regarding ocular screening in children with type 1 diabetes.

#### Graves’ Disease

*Graves’ disease* occurs when the immune cells attack and disrupt the homeostasis of the thyroid gland, leading to hyperthyroidism through the over-production of thyroid hormones. A higher prevalence of Graves’ disease occurs in developed countries, where iodine is readily available. Graves’ disease has an estimated global prevalence of 2 – 3% (93). Graves’ disease has also been associated with other autoimmune disorders, such as myasthenia gravis (94), increasing the risk of ocular manifestations.

Up to 50% of Graves’ disease patients experience ocular manifestations (95), including Graves’ ophthalmology (GO), which occurs in up to a third of patients and can cause symptoms such as diplopia and photophobia (96, 97). Although GO symptoms can occur within months or a few years of the initial diagnosis (8), they can often occur before Graves’ disease diagnosis, again highlighting the eye as an indicator of underlying disease (98). Upper eyelid retraction, dry eye and ptosis are other common symptoms experienced. A positive correlation between increased IOP and age compared to disease severity was also reported (97). In addition, 60% of Graves’ disease patients without ptosis experience increased intraocular pressure upon upward gaze due to enlarged inferior rectus (8).

Vision loss with GO is rare; however the potential occurrence should not be underestimated. Severe complications, such as dysthyroid optic neuropathy account for up to 5% of GO patients (99). Zhang et al. documented the first known case of a GD patient with severe chemosis and conjunctival prolapse. Surgery is usually required to relieve the pressure exerted by enlarged ocular muscles that compress the optic nerve. In this case, orbital radiotherapy provides a safe, effective treatment option as the patient was not a candidate for surgery (100).

#### Hashimoto’s Thyroiditis

*Hashimoto’s thyroiditis* occurs due to immune cells attacking the thyroid and is the primary cause of hypothyroidism in developed countries (101). Has been associated with other autoimmune diseases such as rheumatoid arthritis and type 1 diabetes (102). There is evidence for considerable association of eye problems with Hashimoto’s thyroiditis; however, many large-scale studies are required to allow these initial findings to be made generalizable.

Although thyroid-associated ophthalmopathy (TAO) occurs more commonly with Graves’disease, it has also been reported to occur in 6% of Hashimoto’s thyroiditis patients. Age, disease and smoking status are considered TAO risk factors (103). As TAO has a higher occurrence with Graves’ disease, its association with Hashimoto’s thyroiditis has been neglected in scientific research, with few reports in the literature. Several case studies report severe TAO (104, 105), involving enlargement of ocular muscles. Although rarely, TAO may also present itself in euthyroid patients, indicating the lack of room for complacency when screening patients, as these severe cases can lead to optic nerve compression resulting in visual impairment.

Corneal differences or abnormalities have also been reported in Hashimoto’s patients. Kirgiz et al. observed a Hashimoto’s thyroiditis cohort to have significantly reduced corneal hysteresis and elevated corneal compensated IOP when compared to the control group. Reduction in corneal hysteresis generates pressure on the optic nerve, resulting in complications such as glaucoma (106).

Case studies also report the rare occurrence of severe, long-term diplopia and vision loss in Hashimoto’s patients (107). One patient, previously misdiagnosed with double elevator palsy, experienced severe diplopia over the course of a year. It was not until a positive forced duction test result, blood tests, and ultrasounds indicated hypothyroidism that Hashimoto’s thyroiditis was diagnosed. Diplopia went unresolved with oral prednisolone. This report suggests the importance of regular screening in patients with typical TAO symptoms and atypical symptoms. Inevitable questions arise into if more timely and thorough screening was implemented, would this have avoided misdiagnosis and lead to more successful treatment outcomes?

Dry eye disease occurred in up to 85% of TAO patients (108, 109), with risk factors including proptosis and lowered free thyroxine levels, however, there are few literature reports on the manifestation. Although milder in euthyroid patients, there is no correlation between dry eye disease and Hashimoto’s thyroiditis severity (109). Overall, Hashimoto’s patients have a significantly higher prevalence of dry eye disease *versus* healthy control groups, including substantially lower mean tear production and tear stability. Other reported manifestations included lid retraction, oedema of soft tissues, proptosis and extraocular involvement (110). Within this study, dry eye disease, significantly lower tear production and tear stability, Meibomian gland dysfunction, proptosis and reduced goblet cell density were observed. Mean meibomian gland area loss was 25%, however, it occurred as high as 50% in one patient.

### Dermatological Autoimmune Diseases

Dermatological autoimmune diseases commonly result in patches of irritated or blistered skin all over the body, resulting in discomfort for the patient. Conditions such as systemic sclerosis primarily affect the skin. However, disease involvement in other organs frequently occurs – including in the eye.

#### Psoriasis

*Psoriasis* involves accelerated skin cell growth which results in red, scaly lesions all over the body. Up to 3% of the global population is currently living with psoriasis (111). Prevalence estimations as high as 11.4% have been reported in Norway, and prevalence is increasing (112).

The prevalence of ocular manifestations is disputed, however up to a reported 70% of patients show ocular association (113). A positive correlation between severity of skin exacerbations and ocular manifestations has also been detected (113). Chowdhury et al. found nearly half of screened patients with ocular manifestations to have subtle symptoms or be asymptomatic (114), thus highlighting patients should undergo regular ocular tests regardless of risk factors or if asymptomatic to permit early treatment intervention. In terms of the eye, psoriasis can affect the lid, conjunctiva and cornea (111).

Chronic conjunctivitis affects approximately 64.5% of patients (111, 115, 116). Within the conjunctiva, conjunctival lesions, xerosis, symblepharon, and trichiasis can occur. Chronic blepharitis is also common, resulting in ectropion, trichiasis, madarosis, loss of lid tissue and visual impairment (117), most commonly in younger patients (118). Erbagci et al. found 67.74% of plaque PS patients to have a least one abnormality of the anterior section. Chronic blepharoconjunctavitis was the main ocular presentation in 64.5% of patients. Corneal opacities, cataracts and corneal pigment dispersion were also reported (119).

Dry eye disease occurs in a reported 18.75% of psoriasis patients (111), however, this value varies between studies. Generally, corneal involvement is rare; however, one report outlined peripheral corneal melting syndrome (PCMS) in a patient. In this case, severe corneal ulcer occurred, stemming 90% of the corneal thickness. Despite the potential for vision loss, ocular problems are resolved with aggressive treatment (120).

Uveitis has been reported in up to 20% of patients (121), commonly the pustular form. Associations between psoriasis and DR have been made (122). Furthermore, Ajitsaria et al. reported the first known case of relapsing orbital myositis associated with juvenile psoriasis, which resulted in permanent visual impairment (123).

#### Systemic Sclerosis

*Systemic sclerosis*, or scleroderma, affects up to 2.5 million people worldwide (124), occurring either as a localised form, affecting only the skin or as a systemic form, where multiple organs are affected. In systemic sclerosis, accelerated collagen production results in hard, thick skin developing, as well as swelling and joint pain. Furthermore, scleroderma has also been associated with other autoimmune diseases such as rheumatoid arthritis and Sjogren’s syndrome (125). In the UK alone, there are almost 20,000 people living with systemic sclerosis, with a predicted 26% increase in prevalence by 2038 (126).

Ocular manifestations of varying severity are plentiful in systemic sclerosis, including retinal abnormalities, cataracts, blepharitis, telangiectasia, scleral pits and keratoconus (127, 128). In one study, the most common finding was dry eye disease (64.71%), followed by skin alterations of the eyelids (56.86%) and retinal abnormalities (50.98%). Patients often have reduced vasculature, leading to possible choroidal atrophy (129). In a small scale study carried out by Grennan et al., although dry eye disease and retinal abnormalities were not common amongst these patients, 50% of the patients had choroidal abnormalities involving areas of nonperfusion (130). One patient with keratoconus presented reduced corneal thickness, severe dry eye disease and decreased tear stability in addition to corneal steeping (131). Iris transillumination occurs when scleroderma interferes with iris pigments (132). Scleroderma patients have been found to have thinner subfoveal choroid layers.

Dry eye disease affects over half of systemic sclerosis patients (133). Horan found that although only a third of their cohort experienced dry eye, almost half reported reduced tear secretion, suggesting for potential development to dry eye disease (134). Fibrosis of the lacrimal gland, chronic blepharitis and meibomian gland dysfunction can result in dry eye disease. Previous reports have noted eyelid stiffness occurring in up to 65% of scleroderma patients studied (125), resulting from fibrosis of eyelids. This lid stiffness can result in lagophthalmos, leaving patients vulnerable to damage or infection (132).

Ocular manifestations also occur in the limited disease type. En Coup de Sabre (ECS) has many ocular symptoms, including ptosis, uveitis, dry eye, episcleritis, orbital myositis (135) and reduced size of the globe and ocular muscles (136). The highest known report of ocular symptoms in ECS occurred in 26% of patients. Kertopathy, retinal detachment, restricted eye motility, diplopia, cataracts and corneal astigmatism were observed (136). Although rare, adie pupil has been reported as an initial disease indicator of ECS (135). Linear scleroderma is more common among children (137), however ocular manifestations are more common in the ESC subtype in paediatric patients (138). Linear ECS scleroderma has also been associated with retinal telangiectasia and exudative retinal detachment (137), and uveitis (138).

#### Dermatomyositis

*Dermatomyositis* is the most common form of idiopathic inflammatory myopathy, resulting from the immune system attacking muscle vasculature and connective tissue in the body. Characteristic symptoms include red, scaly skin patches and Gottron papules. Heliotrope rash on the upper eyelid and oedema around the eyes can also occur. Dermatomyositis affects an estimated 9.63 people per million, with females (139) and African Americans (140) at greatest risk. It most commonly affects adults between 40 – 60 and children between 5 – 15, with children generally experiencing more acute disease (141).

Ocular manifestations of dermatomyositis are abundant and include heliotrope rash, oedema surrounding the eyes, ptosis, diplopa, various strabismus, conjunctival oedema, nystagmus, iritis, cotton wool spots, optic atrophy, conjunctival pseudopolyposis, lens abnormalities, episcleritis and uveitis with glaucoma (142). Internuclear ophthalmoplegia has also been reported (143). Irreversible visual impairment as a result of ocular manifestations of dermatomyositis has been reported (144, 145). Systemic methylprednisolone and cyclophosphamide were unable to resolve vision loss caused by damage to the macula and optic disc in a female patient. Again, questions arise surrounding earlier intervention and the potential to avoid permanent visual impairment (144).

Rare cases of acute CRAO associated with DM have been reported in the literature (146), indicated by symptoms such as papilloedema and peripapillary haemorrhages, which cause extensive retinal damage. In this case, ischemia of the optic nerve was deemed responsible for CRAO presentation. Although rare, vision loss with CRAO does occur. In addition, significant irreversible damage occurs for CRAO lasting longer than 240 minutes (147), reinforcing the importance of disease awareness and timely treatment.

Orbital myositis is another rare but severe manifestation of dermatomyositis. The first known report of this occurrence dates back to 2005, where a male patient experienced muscle pain followed by eyelid oedema and ocular pain, coinciding with undiagnosed dermatomyositis. MRI revealed bilateral enlargement of the lateral and inferior rectus muscle, which was successfully treated with immunoglobulin therapy (148).

##### Miscellaneous autoimmune disorders

###### ANCA vasculitis

With the potential to affect various organs within the body, Anti-Neutrophil Cytoplasmic Antibody Associated Vasculitis is primarily characterised by inflammation of blood vessels, leading to their destruction. ANCAs target the cytoplasm of neutrophils, and their attachment results in the initiation of neutrophil attack against blood vessels. This results in swelling and inflammation of blood vessels in various regions of the body. In Europe, between 20 – 25 people per million every year (149).

One such condition that falls under AAV is known as Granulomatosis with polyangiitis (GPA). This condition is characterised by vasculitis of the small and medium blood vessels of several organ systems, including the nose, kidneys and respiratory system. According to the National Organization for Rare Disorders, more significant than half of GPA patients experience ocular symptoms (e.g. conjunctivitis, corneal ulcers, pain, scleral inflammation and vision loss). In some instances, these can appear as the initial symptom of GPA (150).

In a recently published case study, a 47-year-old female did not respond to anti-inflammatory and antimicrobial therapies for the treatment of long term bilateral ocular symptoms (scleral inflammation, secondary glaucoma and corneal ulcer). Corneal ulcer presented as the first symptom of GPA in this instance, later diagnosed through tissue sampling. Additionally, abscessing of the conjunctiva opened to expose the sclera. The patient responded to treatment of cytostatic and prednisolone. However, corneal and conjunctival defects remained, highlighting the potential for long-term, persistent ocular manifestations associated with this autoimmune disease (151).

#### IgG4-Related Disease

Immunoglobulin G4-related disease is a rare and chronic fibro-inflammatory disease characterised by elevated levels of IgE and IgG4. It is a systemic disease that can affect multiple body organs, including the eyes, lungs and kidneys. Whenever the disease affects the eye, it is known as orbital IgG4-related disease, involving the lymphoplasmacytic infiltrations of any adnexal tissues of the eye (e.g. tear glands and eyelids). IgE increases due to overproduction of cytokines, e.g. IL-4 and IL-13, while IgG4 increases due to increased production of IL-10. Overall this results in fibrogenic cytokine TGF-B production, leading to fibrosis (152).

Goto et al. conducted a retrospective multicentre study to assess the clinical symptoms of those with orbital IgG-related disease. Through analysis of 378 patients across nine centres in Japan, imaging studies revealed the occurrence of ocular lesions associated with orbital IgG4-related disease, affecting several ocular tissues, including the eyelids (12%) and, most commonly, the lacrimal gland (86%). Interestingly, lesions of the extraocular tissue occurred in just under half of patients, highlighting that while this is a rare disease, devastating ocular manifestations are frequent in nature. The most common ocular symptom reported was dry eye, occurring in 22% of patients. Further symptoms such as diplopia and reduced vision were also reported (153).

##### Cogan Syndrome

Cogan’s Syndrome is a rare disease, with only approximately 250 reported known cases (154), involving the destruction of tissue in the ear and eyes resulting from immune system attack. The mechanism of this disease is largely unknown; however, patients are known to suffer from ocular symptoms such as irritation and reduced vision as a result of this tissue destruction.

Migliori et al., reported a case study of a 31-year-old female who presented bilateral conjunctivitis alongside ear symptoms, e.g. hearing loss and tinnitus. After 10 days, the patient was unresponsive to initial treatments and their condition worsened, prompting a change in treatment strategy to a systemic corticosteroid. Ten days following initiation of systemic prednisolone, the patient experienced more severe ocular symptoms, including lacrimation and photophobia, amongst other symptoms affecting the ears such as sensorineural hearing loss. Furthermore, interstitial keratitis was detected upon further ophthalmic evaluation, allowing the diagnosis of Cogan’s syndrome. Ocular symptoms dissipated 4 months following initiation of treatment using immunosuppressants (155).

## Epidemiological Factors Affecting the Prevalence of Ocular Complication With Autoimmune Disorders

### Gender Bias in Prevalence of Various Autoimmune Disorders

Within many autoimmune diseases, there is well-documented gender bias in both prevalence and disease severity. In the more than 80 autoimmune disorders that exist, women account for up to an estimated 85% of these patients (156) and are at greater risk of polyautoimmunity (157). Such bias could be influenced by genetics. The X chromosome contains 10% of the body’s miRNA (158). Females see a greater expression of genes such as KDM6A located on the X chromosome, leading to immune responses when abnormalities such as incomplete activation occur (159). Higher expression of transcription factor VGLLL3 in females has also been strongly associated with Sjogren’s syndrome and systemic sclerosis (160) **–** two diseases with female gender bias.

Genetics also influence disease severity. Whereas females generally have a chronic, fibrotic and Th-2 dominant immune response, the characteristic acute inflammation in autoimmune diseases with male bias may result from their predominant Th-1 immune response (161). This may explain why females have higher disease prevalence, yet males often experience more severe disease. This is supported by acute, antibody-mediated diseases such as Graves’ disease and myasthenia gravis being more common amongst women, while acute inflammatory diseases are more common in men.

The independent hormonal milieu of males and females may also contribute. Depending on relevant levels, testosterone and progesterone are reported to have immunosuppressive benefits, whereas oestrogen and prolactin cause the immune response to polarise towards Th2 response and generate higher levels of antibodies (162). Moreover, endocrine transitions women go through in at least two stages of their lives can cause an increased vulnerability to disease predisposition and severity.

#### Rheumatological

Female bias occurs in rheumatological autoimmune diseases such as rheumatoid arthritis and Sjogren’s syndrome. For example, middle-aged women account for over 70% of rheumatoid arthritis cases in most studied patient cohorts (163) and are at greater risk for dry eye disease (139). Alternatively, Bechet’s disease prevalence is higher in males, with a greater risk of ocular manifestations (164).

#### Gastrointestinal

Gender bias in IBD is disputed in the literature. Predisposition between sexes remained relatively equal for ulcerative colitis until the age of 45, where males became more predisposed to the disease (165). Contradictory reports suggest that women have a 20-30% higher risk of developing ulcerative colitis than men (32). Ocular symptoms are more frequent in women with Crohn’s disease (166). Regarding celiac disease, recent studies identified increased risk in undiagnosed celiac disease in girls and women (167), however, Nijhawan et al. suggest higher male incidence (168).

#### Neurological

There is evidence for gender differences in influencing susceptibility to neurological autoimmune disorders. Inflammatory-led multiple sclerosis has a higher female predisposition, however disease progression and severity is worse in males (159). Research suggests pain experienced by female multiple sclerosis patients is due to a more reactive immune system, whereas males experience greater pain manifestation due to rapid neurodegeneration (169).

Myasthenia gravis can occur at any age; however, younger female and older male groups are at greater risk (170). In addition, males are at two times the risk of developing OMG (171).

#### Endocrine

Endocrine autoimmune disorders, such as Hashimoto’s thyroiditis, have a higher prevalence amongst females (102), except type 1 diabetes. As insulin sensitivity is higher in females, this may be one factor accounting for higher disease prevalence amongst males (172). Despite male bias in type 1 diabetes, females experience a 30% higher risk of retinopathy as a manifestation of the disease and increased complications during pregnancy (173, 174).

#### Dermatological

Females are more likely to develop limited scleroderma than males, especially at a younger age. Males tend to instead develop systemic sclerosis at an older age (175). Although gender bias reports are conflicted, recent studies indicate psoriasis is significantly more severe, along with more frequent ocular complications, in males (176). Similarly, although females are at greater risk of dermatomyositis (139), Tseng et al. found that males with dermatomyositis were more likely to experience SS when compared to females (177). Dermatomyositis has a slightly higher female predisposition; however, this declines naturally with age (178).

### Effect of Hormonal Imbalances in the Prevalence of Various Autoimmune Disorders

#### Puberty

Before puberty, sex differences between children are less significant in influencing incidence and severity for both cases due to similarities in a hormonal milieu in this early stage of life. However, less information on childhood autoimmune diseases makes it difficult to identify patterns.

No gender differences are observed for Bechet’s disease prepuberty (178). Similar patterns are seen in young females with MS, with the risk for females doubling *versus* males over the age of 13 (179); however, disease progression and complications occur more rapidly in males. In addition, some diseases increase in severity during puberty, such as type 1 diabetes due to reduced insulin resistance (180). Therefore, physicians should be aware of the potential severe ocular manifestations that could result from a worsened disease state.

#### Pregnancy

Although reports are varied, pregnancy and long-term breastfeeding have been generally found to relieve diseases such as psoriasis (181) and rheumatoid arthritis (182) due to increased anti-inflammatory estradiol levels. Alternatively, pregnancy further increases the risk of developing multiple sclerosis (183).

Although some autoimmune diseases worsen during pregnancy, overall, the body aims to suppress the mother’s immune system to protect the foetus, moving towards a Th2 immune response and thus explaining the periods of remission observed in some autoimmune diseases and their ocular manifestations during pregnancy (184).

#### Menopause

Some diseases, such as rheumatoid arthritis and psoriasis, may worsen following menopause, likely due to the changes in the body’s immune system and higher levels of pro-inflammatory cytokines. Furthermore, men with rheumatoid arthritis were found to have elevated levels of estrogen (185). Mean peak age onset of rheumatoid arthritis coincides with menopausal age, although earlier menopause has been linked with less severe rheumatoid arthritis (186). Some disease courses may not be influenced by menopause. For example, recent data suggest Crohn’s disease and ulcerative colitis severity does not change, and may improve, during menopause (187).

### Geographical and Ethnic Differences

As mentioned in previous sections, susceptibility to specific autoimmune diseases occurs due to genetic predisposition and environmental factors. Epidemiological data suggest apparent ethnic and geographical differences for several of the autoimmune diseases discussed in this review. Overall, African American populations have a higher prevalence of autoimmune diseases. However, there is insufficient information to conclude if this is due to genetic differences, environmental factors, or both. Identifying those most at risk of developing specific autoimmune diseases and thus the associated ocular manifestations can help increase awareness among clinicians and researchers.

#### Rheumatological

Rheumatoid arthritis is more common in Western countries, likely due to environmental factors, for example, smoking being more common. There is little existing information surrounding the prevalence of rheumatoid arthritis in developing countries. In terms of ethnic groups, African American patients are slightly more predisposed to developing rheumatoid arthritis and experiencing lower rates of remission with DMARD treatment (188). In terms of the paediatric rheumatoid arthritis population, there is a higher prevalence of children attending rheumatology clinics in South Asian countries compared to Western countries, except Singapore (189). Most reports cover instances in Western countries; however, this is the first study into South East Asia.

Interestingly, Muro et al. found that levels of anti-NT5C1A, an autoantibody which can be associated with severe disease course, were lower in 314 Japanese patients with systemic sclerosis and Sjogren’s syndrome (190), potentially indicating that Japanese patients could, in general, have less severe disease courses of these diseases. Findings that systemic sclerosis is more common in Europe, Canada and North America support these results. However, more extensive studies are required to determine if this finding can be generalised to the wider population. Furthermore, large scale studies have found that the risk of Sjogren’s to be lower in African American patients (191).

With Bechet’s disease, the highest prevalence is found in the Mediterranean basin (25). In Turkey, the estimated prevalence is 421 per 100,000 population (26). Sub-Saharan Africans are identified to have a more severe disease course (192).

#### Gastrointestinal

The prevalence of Crohn’s disease and ulcerative colitis among different ethnic groups varies between countries. There is little data on disease prevalence in third world countries. Globally, the highest prevalence of Crohn’s disease and ulcerative colitis are seen in Western countries, namely Canada for Crohn’s disease and the USA, Denmark and Iceland for ulcerative colitis (193), and thus most studies originate from these countries. Within a recent UK study, an Indian cohort showed the highest risk of developing ulcerative colitis (194), while other studies show South Asian individuals are at greater risk of both Crohn’s disease and ulcerative colitis (195). Ulcerative colitis is increasing in Asia, signifying that IBD is becoming a global disease rather than a disease in Western countries (196).

In the USA, White and Indian populations are affected more than African American, Hispanic and East Asian populations (197, 198). A study of 228 patients in San Francisco revealed that Asian and Hispanic cohorts were commonly diagnosed at a later age when compared to Black and White cohorts, which could impact disease outcomes and associated ocular manifestation. Furthermore, Crohn’s disease was most common amongst the Black ethnic group compared to the Asian group (199). African Americans are more likely to develop ocular manifestations such as uveitis (193).

As with many autoimmune diseases, the prevalence of the celiac disease has been found to vary within countries. Celiac disease has a high prevalence in North America, North Africa, Middle East and India, yet low prevalence in Sub-Saharan Africa and East Asia (200, 201). For example, a higher prevalence of celiac patients was found in more northern regions of the United States *versus* southern regions (202). Ocular manifestations tend to occur in adult celiac patients opposed to children (203).

#### Neurological

Multiple sclerosis prevalence is higher in North America, Western Europe and Australasia compared to Africa and Oceana (204). In the UK, a large-scale study found the BAME population at greater risk of developing multiple sclerosis compared to Whites, as well as smoking status, despite a similar mean age of diagnosis, with the exception of South Asians (205).

Geographical prevalence of Gullian-Barre Syndrome is influenced by seasonal changes. Overall, incidence of Gullain-Barre syndrome is greater in the winter, particularly in Western countries, the Far East and Middle East. Alternatively, Indian subcontinent and Latin America see reduced incidence in winter (206). Demographics of Gullain-Barre subtypes varies widely throughout the world.

Currently, there are at least 64,000 individuals in the US suffering from myasthenia gravis (76). Research in a South African cohort has shown that while White cohorts are at higher risk of developing myasthenia gravis, Black cohorts are at greater risk of ocular manifestations which are resistant to treatment (207).

#### Endocrinological

The highest prevalence records of type 1 diabetes have been found in European countries, such as Finland, and the lowest rates in South American and Asian countries, such as Venezuela and China (208). Other reports indicate America and Africa have the highest and lowest prevalence, respectively (209). In addition, a large-scale study suggested genetic predisposition to type 1 diabetes was highest in non-Hispanic white individuals (210).

Environmental factors influence the prevalence of Graves’ disease, as countries that are iodine-deficient show higher prevalence (211). There is little information on the association between Graves’ disease and ethnicity. However, in 2014, McLeod et al. first provided evidence that Graves’ disease is more common amongst Black and Asian Pacific Islander populations (212). As developing countries lack comprehensive population-based studies, more research is required to have a specific insight into Graves’ disease, as opposed to hyperthyroidism in general.

Although earlier findings had suggested Hashimoto’s thyroiditis is more common amongst White populations (213), more recent research suggests Japanese and Korean Graves’ disease patients are at greater risk of disease complications when compared to Caucasian people (214). In addition, a study in a South African cohort has shown White populations to be at higher risk of developing myasthenia gravis. Black populations are at greater risk of ocular manifestations which are resistant to treatment (207).

More recent data suggested Caucasians have over 6 times more risk of developing GO (215), while GO is generally less severe in Asian patients (215) and experience less muscle involvement. In Asian populations, complications such as cataracts are more frequent (216).

#### Dermatological

Psoriasis occurs more frequently in Western countries (217), however lack of research in non-Caucasian groups with the disease could contribute to these findings. In America, psoriasis prevalence in Caucasian groups was almost double that of African American and Hispanic groups (218). In particular, one study found psoriasis prevalence to increase with increasing distance from the equator, indicating the influential role of environmental factors such as sunlight in psoriasis. Yan et al. reported that people of colour suffering from psoriasis have a lower quality of life and reduced likelihood of diagnosis (219). As witnessed in case studies, delayed diagnosis and treatment can lead to irreversible vision loss due to underlying ocular manifestations.

A higher prevalence of systemic sclerosis is found in North America and Australia than in Japan and Europe (181). In addition, lower social-economic status can be associated with more severe disease course and manifestations in systemic sclerosis in African American patients (220), confirming the influence of environmental factors in disease severity.

### Drug-Induced Ocular Side Effect in Various Autoimmune Disorders

Along with autoimmune diseases, the drugs used to treat these diseases can also adversely affect the eye. Therefore, clinicians must maintain a delicate balance between effective treatment and minimizing side effects in patients by being aware of those most at risk. Overall, the various therapies used in treating autoimmune diseases are safe and effective. The different common side effects of commonly used drugs to treat autoimmune disorders are listed in **Table 3**.

**Table 3:** Ocular side effects of various drugs used to treat autoimmune diseases.

Drug	Associated Ocular effect	Reference
Methotrexate	Conjunctivitis, dry eye, blepharitis, Cotton wool spots, photophobia, epiphora, optic neuropathy, ocular burning, irritation and blurred vision	(221–223)
Azathioprine	Retinal vasculitis, cytomegalovirus (CMV) retinitis and uveitis	(224)
Hydroxychloroquine	Retinal toxicity, Bull’s eye maculopathy and vortex keratopathy	(225, 226)
Cyclooxygenase inhibitors	Conjunctivitis, blurred vision, branched retinal vein occlusion and thrombosis	(227, 228)
Glucocorticoids	Sub-capsular cataracts, secondary open-angle glaucoma, cataract, optic nerve damage, mydriasis central Verous chorioretinopathy	(229, 230)
Anti-TNFa drugs	Uveitis, vitritis, orbital granuloma, orbital myositis, herpes zoster keratitis, scleritis, optic neuritis and chiasmopathy	(231)
Aminosalicylates	Dry eye, blurred vision, Steven-Johnson syndrome and optic neuropathy	(232, 233)
Psoralen and Ultraviolet A (PUVA)	Conjunctival hyperemia, reduced lens transparency, dry eye, cataract formation and lens opacities	(234, 235, 236)

## Conclusion

Although often undermined and overlooked, all the autoimmune diseases discussed in this review present numerous ocular complications, ranging from minor symptoms to sight-threatening scenarios. Lack of disease awareness, misdiagnosis and untimely treatment intervention can have devastating, permanent effects on patient vision. Overall, the global prevalence of autoimmune diseases is increasing, indicated through epidemiology studies and market reports. This observed rise in numbers is expected to continue due to environmental factors, such as Eastern countries adopting more westernised lifestyles. Genetic factors also influence disease predisposition and severity among many autoimmune diseases, with the majority having female bias. Identifying those at most significant risk to autoimmune diseases and their associated ocular manifestations can help clinicians diagnose, screen, and implement treatment more effectively, even in asymptomatic patients or who show subtle ocular symptoms. The eye can act as an indicator of underlying disease in many cases. Thus clinicians should utilize this important tool and not overlook any minor ocular symptoms occurring in the absence of more obvious disease-specific symptoms.

## Author Contributions

KG: writing - original draft, writing - review & editing. DM: writing - review & editing. TRRS: Project administration, resources, supervision, writing - review & editing. All authors contributed to the article and approved the submitted version.

## Funding

This project is funded by the European Union’s Horizon 2020 research and innovation programme under the Marie Skłodowska-Curie Actions (grant agreement – No 813440). DM is funded by Horizon 2020 Orbital ITN project.

## Conflict of Interest

TR is the Founder and CTO of Re-Vana Therapeutics.

The remaining authors declare that the research was conducted in the absence of any commercial or financial relationships that could be construed as a potential conflict of interest.

## Publisher’s Note

All claims expressed in this article are solely those of the authors and do not necessarily represent those of their affiliated organizations, or those of the publisher, the editors and the reviewers. Any product that may be evaluated in this article, or claim that may be made by its manufacturer, is not guaranteed or endorsed by the publisher.
